# P04 HSD 1624 as an adjuvant of colistin for MDR bacteria

**DOI:** 10.1093/jacamr/dlaf118.011

**Published:** 2025-07-14

**Authors:** Martha Akle Asare, Herman O Sintim, Abiola Isawumi

**Affiliations:** West African Center for Cell Biology of Infectious Pathogens, University of Ghana, Legon, Ghana; University of Notre Dame, Notre Dame, IN, USA; West African Center for Cell Biology of Infectious Pathogens, University of Ghana, Legon, Ghana

## Abstract

**Background and objectives:**

Millions of people die each year from antimicrobial resistance, which is caused by pathogens that are resistant to antibiotics like colistin and carbapenems. Because there are few available treatments, ESKAPE pathogens are classified as critical pathogens (WHO 2020). An excellent substitute is antibiotic-adjuvant research, which has been effectively used to strengthen antibiotics over time. With its mechanism of action being described as affecting both membrane permeability and the production of reactive oxygen species, which leads to bacterial cell lysis, the small molecule compound HSD 1624 may be useful against Gram-negative bacteria. Colistin MICs were also lowered to the breakpoint when used in combination with HSD 1624. However, the effect of this compound on biofilm formation and the expression of resistant markers is unknown. Additionally, its toxicity to immune cells like macrophages and its ability to stimulate the immune system as a strategy to combat AMR needs to be investigated.

**Methods:**

Antibacterial activity was determined using Agar well diffusion assay, time kill assay and micro-broth dilution after which synergy of HSD 1624 with colistin was ascertained with chequerboard assay Its effect on biofilm was determined via biofilm formation and treatment assay. Additionally, its effect on the expression of resistant markers was determined using RT-qPCR to quantify extracted RNA from HSD/ HSD-colistin treated strains and to quantify cytokines produced by induced macrophages through infection and treatment. Host factor interaction was also performed in-vivo to observe HSD 1624 activity in serum.

**Results:**

Findings of this study solidify the claim that HSD 1624 is an effective antibiotic for Gram-negative bacteria and an adjuvant that generally has synergistic effects with colistin. Biofilm strength of the strains used in this study altered after treatment with HSD/HSD-colistin combinations. Additionally, HSD-colistin reduced the expression of resistant markers in strains of interest and induced macrophage (RAW cells) cytokine production after treatment with HSD/ HSD-colistin combination, aiding the successful clearance of Gram-negative bacteria. HSD 1624 was unaffected by serum, effectively eliminating bacteria in-vitro.

**Conclusions:**

This study aims to potentiate last resort antibiotic, colistin against highly resistant Gram-negative Ghanaian clinical isolates. Strategies to combat AMR such as combination therapy, drug re-purposing and immune stimulation were employed against these strains in question. Findings of this study can be applied in antibiotic potentiation studies, aiding preclinical and clinical studies on HSD 1624.

